# Cytosolic p53 Inhibits Parkin-Mediated Mitophagy and Promotes Acute Liver Injury Induced by Heat Stroke

**DOI:** 10.3389/fimmu.2022.859231

**Published:** 2022-05-13

**Authors:** Wei Huang, Weidang Xie, Hanhui Zhong, Shumin Cai, Qiaobing Huang, Youtan Liu, Zhenhua Zeng, Yanan Liu

**Affiliations:** ^1^Department of Critical Care Medicine, Nanfang Hospital, Southern Medical University, Guangzhou, China; ^2^The First School of Clinical Medicine, Southern Medical University, Guangzhou, China; ^3^Department of Anesthesiology, Affiliated Hospital of Guangdong Medical University, Zhanjiang, China; ^4^Guangdong Provincial Key Lab of Shock and Microcirculation, Department of Pathophysiology, Southern Medical University, Guangzhou, China; ^5^Department of Anesthesiology, Shenzhen Hospital, Southern Medical University, Shenzhen, China

**Keywords:** mitophagy, acute liver injury, p53, Parkin, apoptosis

## Abstract

Heat stroke (HS) is a severe condition characterized by increased morbidity and high mortality. Acute liver injury (ALI) is a well-documented complication of HS. The tumor suppressor p53 plays an important role in regulation of mitochondrial integrity and mitophagy in several forms of ALI. However, the role of p53-regulated mitophagy in HS-ALI remains unclear. In our study, we discovered the dynamic changes of mitophagy in hepatocytes and demonstrated the protective effects of mitophagy activation on HS-ALI. Pretreatment with 3-MA or Mdivi-1 significantly exacerbated ALI by inhibiting mitophagy in HS-ALI mice. Consistent with the animal HS-ALI model results, silencing Parkin aggravated mitochondrial damage and apoptosis by inhibiting mitophagy in HS-treated normal human liver cell line (LO2 cells). Moreover, we described an increase in the translocation of p53 from the nucleus to the cytoplasm, and cytosolic p53 binds to Parkin in LO2 cells following HS. p53 overexpression using a specific adenovirus or Tenovin-6 exacerbated HS-ALI through Parkin-dependent mitophagy both *in vivo* and *in vitro*, whereas inhibition of p53 using siRNA or PFT-α effectively reversed this process. Our results demonstrate that cytosolic p53 binds to Parkin and inhibits mitophagy by preventing Parkin’s translocation from the cytosol to the mitochondria, which decreases mitophagy activation and leads to hepatocyte apoptosis in HS-ALI. Overall, pharmacologic induction of mitophagy by inhibiting p53 may be a promising therapeutic approach for HS-ALI treatment.

## Introduction

Heat stroke (HS) is the most serious heat-related illness, defined as having elevated core body temperature (Tc) >40°C with central nervous system dysfunction (1). Recently, HS incidence rates have increased, especially with the growing frequency of heatwaves (2). Of note, HS is associated with multiple organ dysfunction syndrome (MODS), increased morbidity, and high mortality due to the combined effects of heat cytotoxicity, coagulopathies, and systemic inflammatory response syndrome (SIRS) (3). Acute liver injury (ALI) is a well-documented complication of HS and is strongly predictive of mortality (4). Importantly, the initial dysregulation of hepatocellular function has been associated with problematic microcirculation and a cytokine storm during early progression of HS (5). Massive pathological changes in hepatocytes are directly correlated with the pathogenesis of heat stroke, including cell death and the inflammatory response (6, 7). However, the mechanisms mediating HS-induced hepatocyte injury remain largely unexplored.

Mitochondria are both primary sources and targets of reactive oxygen species (ROS), and mitochondrial dysfunction is a hallmark of HS-mediated pathology (8). Increased oxidative injury following HS exacerbates mitochondrial compromise, and subsequent resultant mitochondrial inefficacy could cause additional increased ROS levels (9). Notably, mitophagy has been identified as an important response of self-regulating mitochondrial quality control and mitochondrial ROS by selectively removing impaired mitochondria (10). Furthermore, mitophagy is the most important mechanism for removing dysfunctional mitochondria, its dysfunction may result in accumulation of damaged mitochondria, release of excessive ROS, and activation of apoptosis-inducing factors (11). PTEN-induced putative kinase 1 (PINK1)-parkin RBR E3 ubiquitin protein ligase (Parkin)-mediated mitophagy is a Parkin-dependent pathway of mitophagy and one of the most recognized mitophagy pathways (12). When mitochondria are impaired and lose their membrane potential, PINK1 accumulates in the outer mitochondrial membrane and then recruits and phosphorylates ubiquitin and the E3 ubiquitin ligase Parkin, leading to the ubiquitination of various mitochondrial proteins, including the outer membrane proteins VDAC1 and mitofusin 1/2 (Mfn1/2) (13). This modification recruits the autophagy adaptor, such as molecule sequestosome 1 (p62/SQSTM1), Nuclear Domain 10 Protein 52 (NDP52), and Optineurin (OPTN), then they bind LC3 protein on autophagosomal membranes, ultimately triggering selective removal of damaged mitochondria by mitophagy (14).

p53 has been well characterized for its response to different cellular stresses, including induction of tumor suppression, growth arrest, senescence, and cell death (15). Upregulation of p53 is involved in triggering HS-induced apoptosis (16). Moreover, recent research found that p53 binds to Parkin’s really interesting new gene 0 (RING 0) region, which impairs the removal of damaged mitochondria by blocking Parkin mitochondrial translocation (17, 18). Parkin has been shown to have important roles in the induction of mitophagy, labeling damaged mitochondria for autophagic removal *via* its ubiquitin ligase activity (19). Other recent studies also indicated that binding of cytosolic p53 to Parkin inhibits mitochondrial translocation of Parkin and activation of Parkin’s E3 ubiquitin ligase, consequently disrupting Parkin-mediated mitophagy (20, 21). A previous study also demonstrated that the PINK1-Parkin-dependent pathway of mitophagy was increased in HS-induced hypothalamic injury (22). However, the role of Parkin-mediated mitophagy in HS-ALI remains largely unknown.

The purpose of this study was to explore the involvement and regulation of mitophagy in HS-ALI. We further attempted to determine the effect of p53-Parkin coregulation of mitophagy on mitochondrial damage and apoptosis in HS-ALI. Our study reveals critical roles for p53 and Parkin in HS-induced mitophagy and provides an opportunity for developing new therapeutics for HS.

## Materials and Methods

### Animals

C57BL/6 mice (aged 10 to 12 weeks) used in this study were obtained from the Experimental Animal Center of Southern Medical University. All mice were housed under a controlled 12/12-h light/dark cycle at a constant temperature (24 ± 1°C) and (54 ± 2%) relative humidity with free access to a pelleted rodent diet and water. All protocols of animal experiments followed the guidelines approved by the Chinese Association of Laboratory Animal Care and were approved by the Ethical Committee for Animal Experimentation of Nanfang Hospital.

### HS Protocol for Animals

HS-ALI models were induced in mice as previously described in our reported method (23). Animals were placed in a climate chamber that was maintained at a constant temperature of 39.5 ± 0.2°C with 60 ± 5% relative humidity in the absence of food or water, and rectal temperature (Tc) was measured at intervals of ten minutes. The time point at which the Tc reached 42.5°C was used as a reference point of HS onset. All mice were then returned to their original cages in an environment at 25°C with water after HS. The control group underwent the same procedure without HS treatment. Chemical reagents at doses of 20 mg/kg for 3-methyladenine (3-MA), 25 mg/kg for Mdivi-1, 25 mg/kg for Tenovin-6, or 2.2 mg/kg for pifithrin-α (PFT-α) were intraperitoneally injected 2 h before HS as required. All groups of mice were sacrificed at time points as indicated after 1.0% pentobarbital sodium (5 mg/100 g.BW, i.p.) administration to harvest the serum and liver tissues.

### Cell Culture, Treatment, and Transfection With siRNA or Adenovirus

Normal human liver (LO2) cells were purchased from the Laboratory of iCell Biotechnology Company (Shanghai, China). LO2 cells were cultured in Dulbecco’s modified Eagle’s medium (DMEM) with 10% fetal bovine serum (FBS). LO2 cells were maintained in a humidified CO_2_ incubator at 37°C. For HS treatment *in vitro*, the culture medium was replaced with fresh medium, and then cells were placed in an incubator containing 5% CO_2_ at 42 ± 0.5°C for 3 h. Subsequently, the cells were incubated in a normal incubator at 37°C and 5% CO_2_. For treatment with PFT-α, cells were pretreated with PFT-α (20 µM, Sigma-Aldrich) 2 h before HS treatment. Lipofectamine 2000 reagent (L3000008, Thermo Fisher Scientific) was used to transiently transfect LO2 cells with short interfering RNA (siRNA) oligonucleotides against Parkin, p53, and negative control siRNA. The siRNAs were synthesized by Gene Pharma (Shanghai, China), and the sequences of siRNA oligonucleotides were as follows: Parkin siRNA 5′-GAGUAGCCG CAAAUGUGCUUCAUCU-3′, p53 siRNA 5′-UCCAGCUCAAGGAGGU GGUUGCUAA-3′; negative control siRNA, 5′-UUCUCCGAACGUCACGU-3’. After cells were transfected for 72 h, subsequent experiments were performed. The adenoviral vector expressing Flag- and GFP-tagged wild-type p53 (Ad-p53) was provided by Gene Pharma (Shanghai, China).

### Western Blot Analysis

Cytoplasmic and nuclear fractions of LO2 cells were obtained using a Nuclear and Cytoplasmic Protein Extraction kit (Beyotime Biotechnology, China). For whole‐cell lysates, liver tissues and LO2 cells were harvested using radioimmunoprecipitation assay (RIPA) lysis buffer containing 1× protease inhibitor cocktail. After separation by sodium dodecyl sulfate (SDS)-polyacrylamide gel electrophoresis, the proteins were transferred to polyvinylidene difluoride (PVDF) membranes. Subsequently, the membranes were blocked in 5% bovine serum albumin (BSA) at room temperature (RT) for 1 h followed by immunoblotting at 4°C overnight with primary antibodies. The membranes were then incubated with secondary antibodies for one hour. Target protein was detected using enhanced chemiluminescence reagents. The primary antibodies were as follows: anti-Parkin (4211, Cell Signaling), anti-PINK1 (6946, Cell Signaling), anti-p53 (60283-2, Proteintech), anti-SQSTM1/p62 (A7758, ABclonal), anti-LC3-II/I (12741, Cell Signaling), anti-LaminB1 (23498-1-AP, Proteintech), anti-BAX (50599-2-Ig, Proteintech), anti-Bcl2 (12789-1-AP, Proteintech), anti-GAPDH (AC002, ABclonal), and anti-VDAC1 (55259-1-AP, Proteintech). The secondary antibodies were horseradish peroxidase-labeled goat anti-mouse (AS014, ABclonal) and anti-rabbit (AS003, ABclonal) antibodies. Quantification was performed by measuring the density of blot bands using ImageJ software. Protein expression levels were normalized relative to the levels of VDAC, LaminB1, or GAPDH.

### Histopathology and Immunofluorescence Staining

Fresh liver tissues were fixed in 10% formaldehyde, dehydrated, embedded in paraffin, sectioned, and stained with hematoxylin and eosin (H&E). H&E staining of livers was assessed under an optical microscope (Zeiss, Thuringia Germany). Liver injury scores were evaluated in 8 randomly selected, nonoverlapping fields at 200× magnification in each section. The sections were analyzed by two professional pathologists who were blinded to the experimental protocol and scored the liver damage.

To visualize the mitochondria, cells were cultured on cover slips and then incubated using a MitoTracker Red fluorescent probe kit (300 nM at 37°C for 30 minutes; Invitrogen, Waltham, USA). For immunostaining, the cells were fixed in 4% paraformaldehyde for 15 minutes and permeabilized with 0.1% Triton X-100 at RT. Cells were incubated with the specific primary antibody anti-LC3-II/I (12741, Cell Signaling) at 4°C overnight followed by incubation with a fluorescent secondary antibody. After counterstaining with 4’ 6-diamidino-2-phenylindole (DAPI), the slides were observed using a confocal inverted laser microscope (Zeiss, Germany).

### Mitochondrial Membrane Potential (JC-1) Assay

Mitochondrial membrane potential (ΔΨm) was measured by monitoring the fluorescent aggregates of JC-1 using a JC-1 detection kit (Beyotime Biotechnology, China) according to the manufacturer’s instructions. We washed cells with PBS buffer three times and prepared the JC-1 fluorescent probe. After staining at 37°C for 30 minutes, relative fluorescence was analyzed by flow cytometry (BD Dickinson, USA).

### Detection of Apoptosis and Intracellular ROS

Apoptosis was assessed using the Annexin V-FITC Apoptosis Detection Kit (BestBio, China). Cells were collected and resuspended in binding buffer containing Annexin V-FITC. After a 15-min incubation at RM in darkness, the buffer was degraded by centrifugation, and the cells were then resuspended in propidium iodide (PI) solution for 10 minutes. Then, the cells were immediately analyzed using flow cytometry (Becton Dickinson, USA) to quantify levels of apoptosis. Annexin V (+)/PI (-) and Annexin V (+)/PI (+) cells in the right quadrant were considered apoptotic.

Cells were pretreated with Parkin siRNA or PFT-α and then cultured at 42°C for 3 h. After staining with 2’-7’-dichlorofluorescin diacetate (Beyotime Institute of Biotechnology) at 37°C for 20 minutes in the dark, relative fluorescence was measured by flow cytometry (BD Dickinson, USA).

### Mitochondria Protein Extraction

Mitochondrial and cytosolic fractions were isolated from LO2 cells using the Cell Mitochondria Isolation Kit (Beyotime Biotechnology, China) according to the manufacturer’s protocols. Lysed cells were resuspended in mitochondrial isolation reagent and centrifuged at 600 × g at 4°C for 10 minutes to obtain the supernatant. Supernatants were then centrifuged at 13,000 g for 5 min to obtain a pellet containing the mitochondria. Protein concentrations were quantified using a bicinchoninic acid (BCA) assay (Beyotime Biotechnology, China) and stored at -80°C.

### Liver Function Assessment

Blood samples were collected 12 h after HS and then centrifuged at 3,000 rpm for 10 min at RT to obtain the serum. Plasma alanine aminotransferase (ALT) and aspartate aminotransferase (AST) levels were analyzed using an automatic biochemical analyzer (Chemray 240, Shenzhen, China).

### Caspase 3 Activity Assay

Caspase 3 activity in cytosolic extracts was determined using the Caspase 3 Activity Assay kit (Beyotime Biotechnology, China) according to the manufacturer’s protocols. Briefly, supernatants from cell lysates were treated with a fluorogenic substrate of caspase 3, Ac-DEVE-MCA, at 37°C for 30 mins. Caspase 3 activity is represented as the relative cumulative fluorescence of the kinetic reaction and is expressed as the relative value normalized to untreated controls, which was measured using a microplate reader (Multiskan MK3, Thermo) at 405 nm.

### Transmission Electron Microscopy

Fresh liver tissues of 1 mm^3^ were harvested and then fixed in 2% formaldehyde and 2.5% glutaraldehyde. The tissue blocks were then fixed in 1% osmic acid, dehydrated with ethanol and acetone gradients, and embedded in epoxy resin and propylene oxide overnight. Samples were then cut into 70-nm-thick ultrathin sections. The sections were then observed under an H-7650 transmission electron microscope (Hitachi, Tokyo, Japan) at low magnification (×8,000) and then analyzed at high magnification (×40,000) to observe mitochondrial autophagosomes and autophagolysosomes. Two professional pathologists analyzed the images in a blinded manner.

### Co-Immunoprecipitation (Co-IP) Assays

Cells were pretreated with 5 mM MG132, a proteasome inhibitor, before exposure to HS. Cell lysates were incubated with 4 µg anti-Parkin or anti-VDAC antibody overnight at 4°C. Then, 50 µl of protein A+G agarose beads was added and centrifuged at 4°C for 3 h. After centrifugation, the supernatant was discarded to obtain immunocomplexes, and the beads were then resuspended in 1× lysis buffer for western blotting analysis using the indicated antibodies. IgG served as the negative control.

### Statistical Analysis

Results are expressed as the mean ± standard deviation. Student’s t test was used to analyze differences between two groups. Statistical comparisons of three or more groups were performed using one-way analysis of variance (ANOVA), followed by *post hoc* analysis. Quantitative data are from at least three separate experiments performed in duplicate. All tests for statistical significance were performed using GraphPad software (La Jolla, CA). P values < 0.05 were considered statistically significant.

## Results

### Mitophagy Is Induced by HS *In Vivo* and *In Vitro*

**Figure 1:**
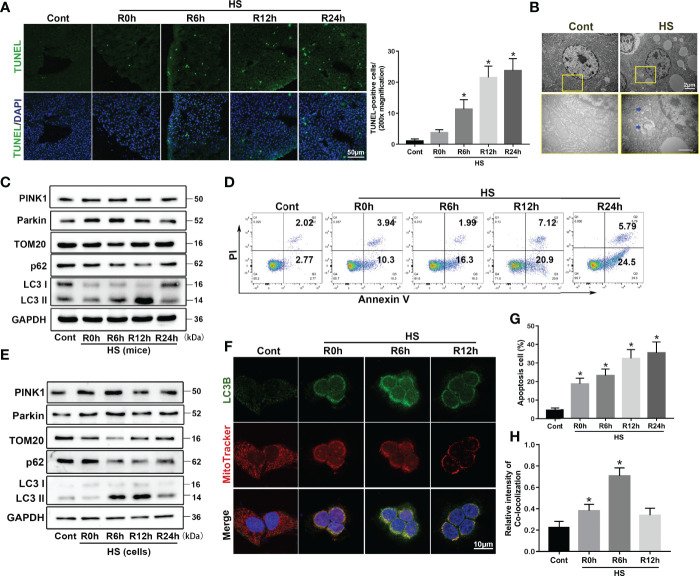
Parkin-mediated mitophagy is induced by HS *in vivo* and *in vitro*. Mitophagy is induced by HS-ALI *in vivo* and *in vitro*. Liver tissues were obtained from HS and sham control mice at the time point of HS onset (0 h) and 6 h, 12 h, and 24 h later. LO2 cells were cultured at 42°C for 3 h to simulate HS followed by incubation at 37°C for 0, 6, 12, and 24 h **(A)** Apoptosis observed by TUNEL staining of liver tissues with quantification of TUNEL-positive cells. Scale bar: 50 μm. **(B)** Representative TEM images of mitochondrial morphology in hepatocytes at 6 h following HS. Blue arrows: mitophagosome and mitolysosome. Scale bar: 2 μm. **(C)** Immunoblot analysis of mito/autophagy-related proteins, including PINK1, Parkin, TOM20, p62, and LC3-II, in liver tissues following HS. **(D, G)** Apoptosis was assessed using Annexin V-FITC/PI staining. **(E)** Immunoblot analysis of mito/autophagy-related proteins, including PINK1, Parkin, TOM20, p62, and LC3-II in HS-induced LO2 cells. **(F, H)** Representative images and quantification of immunofluorescence double-labeling LC3B (green) and MitoTracker (red) in HS-treated LO2 cells, scale bar: 10 μm. Data are shown as the mean ± SD. n = 4. *p < 0.05.

To clarify the role of mitophagy in HS-ALI, we first demonstrated the occurrence of mitophagy in the liver following HS. As shown in **Figure 1A**, TUNEL staining revealed an increase in TUNEL-positive cells following HS treatment in a time-dependent manner, indicating the occurrence of apoptosis compared to the controls. TEM revealed mitochondrial swelling, loss of mitochondrial cristae, and formations of mitophagosomes and mitophagolysosomes in hepatocytes in HS-treated mice, which were rarely observed in controls (**Figure 1B**). Immunoblot analysis of the mitophagy biomarkers Parkin and PINK1 gradually increased at 0-6 h, and returned to baseline by 24 h; mitochondrial protein Translocase of outer mitochondrial membrane 20 (TOM20) gradually decreased at 0-6 h, and returned to baseline by 24 h. In contrast, p62 expression reached the lowest levels at 12 h after HS, and LC3-II expression gradually increased at 6-12h then slightly decreased following HS (**Figures 1C**, **S1A**), suggesting that mitophagy was induced in HS-ALI mice.

Compared to the control group, flow cytometry analysis showed an increase in apoptotic cells following HS in a time-dependent manner, indicating that apoptosis was induced in LO2 cells by HS (**Figures 1D, G**). The mitophagy biomarkers Parkin and PINK1 gradually increased at 0-6 h, and returned to baseline by 24 h; mitochondrial protein TOM20 gradually decreased at 0-6 h, and returned to baseline by 24 h. In contrast, p62 expression reached the lowest levels at 12 h after HS, and LC3-II expression gradually increased at 6-12 h then slightly decreased following HS (**Figures 1E**, **S1B**). Interestingly, immunoblot analysis indicated an increase of PINK1 expression levels in the mitochondria at 6 h after HS, and an increase in the translocation of Parkin from the cytoplasm to the mitochondria *in vivo* and *in vitro* (**Figures S1C, D**), suggesting that HS stabilized PINK1 at the outer mitochondrial membrane and thereby recruits more Parkin to mitochondria. To further evaluate HS-induced mitophagy, we used double staining of LC3B and MitoTracker, a mitochondrial marker, in HS-treated LO2 cells. Confocal imaging revealed that staining for the autophagosome marker LC3B was distinctively colocalized with MitoTracker at 0-6 h following HS, indicating the formation of mitophagosomes induced by HS (**Figures 1F, H**). Collectively, these findings indicate that mitophagy is temporarily enhanced and then continues to decline in HS-ALI.

### Mitophagy-Deficiency Increases Apoptosis in HS-ALI

**Figure 2:**
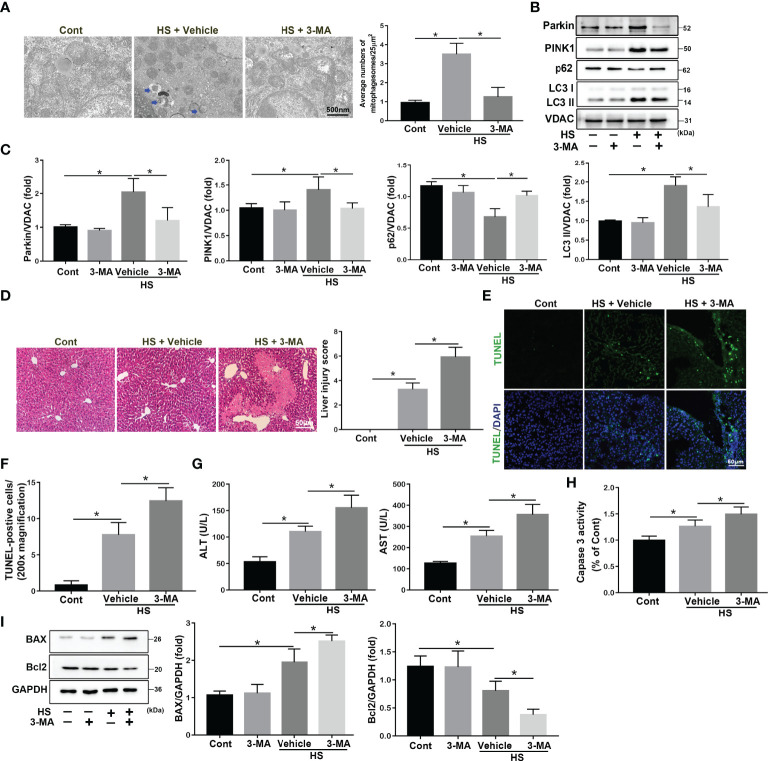
3-MA treatment increases apoptosis in HS-ALI. Mice were pretreated with 3-MA (20 mg/kg, i.p.) for 2 h and then subjected to sham-untreated or HS. Livers and serum were collected 6 hours after HS. **(A)** Representative TEM images of mitochondrial morphology in hepatocytes after HS. Blue arrows: mitophagosome and mitolysosome. Scale bar: 500nm. **(B, C)** Immunoblot analysis and quantification of mito/autophagy-related proteins, including Parkin, PINK1, p62, and LC3-II, in the mitochondrial fractions of livers. **(D)** Representative histology and pathological score of liver samples by H&E staining. Scale bar: 50 μm. **(E, F)** Apoptosis was observed by TUNEL staining of liver tissues and quantification of TUNEL-positive cells. Scale bar: 50 μm. **(G)** Relative serum ALT and AST levels. **(H)** The enzymatic activity of Caspase 3 was subsequently measured. **(I)** Immunoblot analysis and quantification of BAX and Bcl2 in liver tissues. n = 4. Data are shown as the mean ± SD. n = 4. *p < 0.05.

Mitophagy was reported to be essential for selectively eliminating damaged mitochondria through autophagy (24). To explore the role and regulation of mitophagy in acute liver damage in HS-ALI mice, the autophagy inhibitor 3-MA was used to inhibit the autophagic process of damage mitochondria clearance. TEM examinations revealed that 3-MA inhibited the formation of autophagosomes in hepatocytes and exacerbated mitochondrial damage, as demonstrated by the presence of fragmentation, swelling, and vacuoles in the mitochondrial matrix, which further aggravated acute liver injury in response to HS (**Figure 2A**). Furthermore, 3-MA significantly decreased mitophagy, as indicated by the downregulation of mitophagy-related proteins in mitochondria in the liver tissues (**Figures 2B, C**). 3-MA pretreatment significantly exacerbated acute liver damage and increased apoptosis in HS-treated mice, as evidenced by increased liver histological scores, TUNEL-positive cells, Caspase 3 activity, and serum ALT and AST levels (**Figures 2D–H**). Two major proteins of intrinsic apoptosis pathway are apoptosis-related proteins BCL-2 Associated X Protein (BAX) as a proapoptotic protein and B-cell lymphoma 2 (Bcl2) as an antiapoptotic one (25). Correspondingly, 3-MA significantly exacerbated apoptosis in HS-induced mice, as evidenced by the upregulate of BAX and downregulate of Bcl2 levels (**Figure 2I**). In agreement with these results above, Mdivi-1, a dynamin-related protein 1/mitophagy inhibitor, significantly inhibited mitophagy as indicated by western blotting of mito/autophagy-related proteins as well as the formation of autophagosome containing damaged mitochondria (**Figures S2A–C**). Mdivi-1 also exacerbated apoptosis in HS-induced mice, as shown by western blotting of apoptosis-related proteins and Caspase 3 activity (**Figures S2D–H**). Therefore, these results suggest that mitophagy-deficiency exacerbates hepatocyte apoptosis in HS-ALI mice.

### Parkin Deficiency Exacerbates Apoptosis and Mitochondrial Damage in HS-Induced LO2 Cells

**Figure 3:**
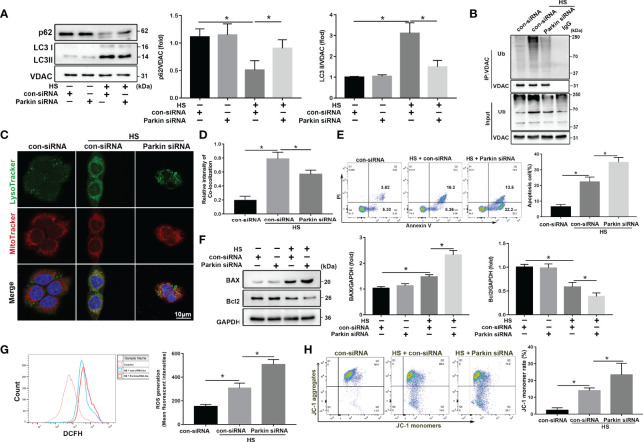
Parkin deficiency exacerbates apoptosis and mitochondria damage in HS-induced LO2 cells. After transfection with con-siRNA or Parkin siRNA for 8 h, LO2 cells were exposed to 42°C for 3 h and then incubated at 37°C for 6 h **(A)** Immunoblot analysis and quantification of mito/autophagy-related proteins, including Parkin, p62, and LC3-II, in the mitochondrial fractions of LO2 cells. **(B)** Immunoprecipitation (IP) analysis of VDAC ubiquitination levels. **(C, D)** Representative images and quantification of immunofluorescence double-labeling LysoTracker (green) and MitoTracker stains (red) in HS-treated LO2 cells. Scale bar: 5 μm. **(E)** Apoptosis was assessed using Annexin V-FITC/PI staining. **(F)** Immunoblot analysis and quantification of BAX and Bcl2 in HS-treated LO2 cells. **(G)** Representative images and quantification of the ROS probe DHE staining in HS-treated LO2 cells. **(H)** Flow cytometry analysis of ΔΨm in LO2 cells stained with JC-1. The percentage represents LO2 cells with damaged mitochondria (low ΔΨm). Data are shown as the mean ± SD. n = 4. *p < 0.05.

To determine the role and function of Parkin-mediated mitophagy in HS-treated LO2 cells, siRNA was used to silence Parkin. siRNA transfection successfully inhibited protein levels of Parkin in LO2 cells (**Figure S3A**). Parkin knockdown dramatically reduced mitophagy activation by p62 and LC3-II in mitochondria in LO2 cells following HS (**Figure 3A**). Parkin knockdown also decreased the ubiquitination levels of VDAC (**Figure 3B**). In addition, colocalization of LC3B and MitoTracker revealed reduced formation of mitophagosomes after silencing Parkin (**Figures 3C, D**), suggesting that Parkin-dependent mitophagy was activated by HS and then prevented by silencing Parkin. Furthermore, silencing Parkin remarkably exacerbated apoptosis in LO2 cells following HS, as shown by western blotting and Caspase 3 activity assays (**Figures 3E, F**, **S3B**). Taken together, these data suggested that Parkin-mediated mitophagy deficiency exacerbates hepatocyte apoptosis after HS.

Increased ROS release is positively correlated with cell death and pathological disorders (26). Oxidative stress levels, as assessed using the ROS probe DHE, was agitated by HS and further exacerbated by silencing Parkin (**Figure 3G**). We measured changes in ΔΨm using JC-1 staining. As shown in **Figure 3H**, HS significantly decreased ΔΨm, which was further exacerbated by silencing Parkin. Together, these data demonstrate that Parkin-mediated mitophagy deficiency may exacerbate HS-induced mitochondrial damage.

### Cytosolic p53 Binds to Parkin Following HS

**Figure 4:**
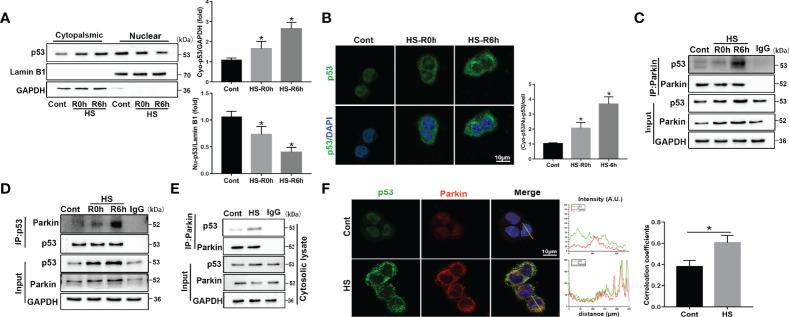
Translocation of p53 from the nucleus to the cytoplasm and cytosolic p53 binding to Parkin following HS. LO2 cells were exposed to 42°C for 3 h for HS treatment and then incubated at 37°C for 0 or 6 h **(A)** Immunoblot analysis and quantification of p53 in the nucleus and cytoplasm after HS. **(B)** Representative images of immunofluorescence labeling of p53 in the nucleus and cytoplasm in HS-treated LO2 cells with quantification of the ratio of fluorescence intensity of cytoplasmic p53 to nuclear p53 in LO2 cells. Scale bar: 10 μm. **(C, D)** Endogenous Parkin-p53 complex in total lysates of HS-treated LO2 cells. **(E)** The endogenous Parkin-p53 complex in cytosolic lysates of LO2 cells following HS. **(F)** Representative images and quantification of immunofluorescence double-labeling p53 and Parkin in HS-treated LO2 cells. Scale bar: 10 μm. Data are shown as the mean ± SD. n = 4. *p < 0.05.

p53 is reported to be an important molecule for the crosstalk of autophagy and apoptosis (27). Cytosolic p53 has been shown to bind to Parkin and disrupt the clearance of damaged mitochondria by Parkin-mediated mitophagy in diabetic islets (28). Other recent studies also reported that p53 exerts distinct cellular functions depending on its cellular concentration and distribution (29, 30). Here, we found that p53 protein levels gradually increased after HS compared to the controls *in vivo* and *in vitro* (**Figures S4A, B**). To further assess whether the intracellular distribution of p53 was altered and whether cytosolic p53 binds to Parkin in HS-induced LO2 cells, immunoblotting analysis and immunofluorescence staining were used. Nuclear p53 protein levels were progressively reduced, and cytoplasmic levels of p53 gradually increased, suggesting an increase in the translocation of p53 from the nucleus to the cytoplasm at 0-6 h following HS (**Figures 4A, B**). In particular, the endogenous Parkin-p53 complex was identified in immunoprecipitants of endogenous Parkin and p53, as well as in the cytosolic lysate of LO2 cells after HS (**Figures 4C–E**). Moreover, colocalization of p53 and Parkin suggested that p53 could bind to Parkin in HS-treated LO2 cells, as indicated by immunofluorescence staining (**Figure 4F**). These data demonstrate that cytosolic p53 binds to Parkin in HS-treated LO2 cells.

### p53 Regulates Parkin-Dependent Mitophagy and Apoptosis in HS-ALI

**Figure 5:**
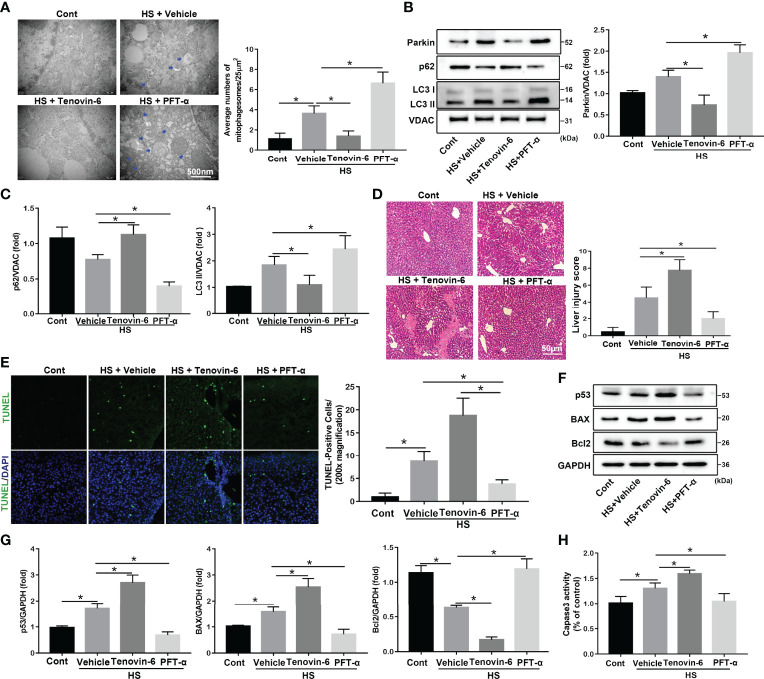
p53 regulates Parkin-dependent mitophagy and apoptosis in HS-ALI. Mice were pretreated with the selective p53 agonist Tenovin-6 (25 mg/kg, i.p.) or the p53 inhibitor PFT-α (2.2 mg/kg, i.p.) for 2 h and then subjected to HS, these livers and blood were collected 6 hours after HS. **(A)** Representative TEM images of mitochondrial morphology in hepatocytes after HS. Blue arrows: mitophagosome and mitolysosome. Scale bar: 500 nm. **(B, C)** Immunoblot analysis and quantification of mito/autophagy-related proteins, including Parkin, p62, and LC3-II, in the mitochondrial fractions of livers. **(D)** Representative histology and pathological score of liver samples by H&E staining. Scale bar: 50 μm. **(E)** Apoptosis was observed by TUNEL staining of liver tissues and quantification of TUNEL-positive cells. Scale bar: 50 μm. **(F, G)** Immunoblot analysis and quantification of p53, BAX, and Bcl2 proteins in liver tissues. **(H)** The enzymatic activity of Caspase 3 was determined. Data are shown as the mean ± SD. n = 4. *p < 0.05.

Crosstalk between cytosolic p53 and Parkin-dependent mitophagy has been reported (31). To investigate the role of p53 in regulating mitophagy in HS-ALI, the p53 agonist Tenovin-6 and the selective p53 inhibitor PFT-α were used in HS-ALI mice. TEM revealed that p53 activation by Tenovin-6 prevented the formation of mitochondrial autophagosomes in hepatocytes and exacerbated mitochondrial damage (**Figure 5A**). Correspondingly, Tenovin-6 pretreatment significantly inhibited mitophagy, as indicated by the downregulation of Parkin, LC3-II, and upregulation of p62 protein expression in mitochondria in the livers of HS-induced mice (**Figures 5B, C**). In addition, Tenovin-6 exacerbated HS-ALI, as evidenced by increased liver histological scores, TUNEL-positive cells, and serum ALT and AST levels (**Figures 5D, E**, **S4C, D**). Tenovin-6 also exacerbated HS-induced apoptosis in HS-ALI mice (**Figures 5F–H**). In contrast, the p53 inhibitor PFT-α attenuated apoptosis in HS-ALI by improving Parkin-dependent mitophagy (**Figures 5A–H**). Collectively, these data suggest that p53 regulates Parkin-dependent mitophagy and apoptosis in HS-ALI.

### p53 Regulates Parkin-Dependent Mitophagy and Apoptosis in HS-Treated LO2 Cells

**Figure 6:**
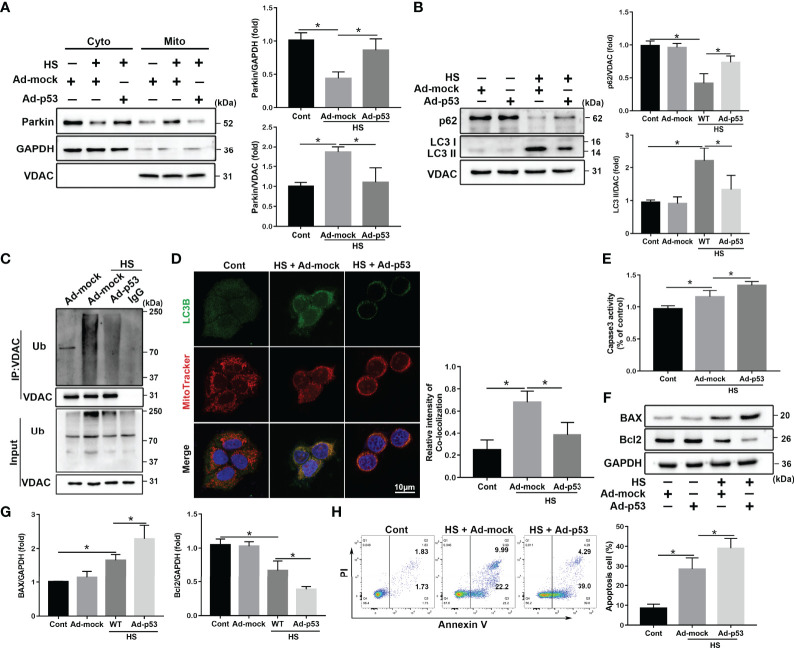
p53 overexpression exacerbates apoptosis through Parkin-dependent mitophagy *in vitro*. After transfection with the empty adenovirus (Ad-mock) or p53 adenovirus (Ad-p53) Ad-p53 for 8 h, LO2 cells were exposed to 42°C for 3 h for HS treatment followed by incubation at 37°C for 6 h **(A)** Immunoblot analysis and quantification of Parkin in the cytoplasm and mitochondrial fractions after HS. **(B)** Immunoblot analysis and quantification of autophagy-related proteins p62 and LC3-II in the mitochondrial fractions of LO2 cells. **(C)** Immunoprecipitation analysis of VDAC ubiquitination levels. **(D)** Representative images and quantification of immunofluorescence double-labeling LC3B (green) and MitoTracker stains (red) in HS-treated LO2 cells. Scale bar: 10 μm. **(E)** The enzymatic activity of Caspase 3 was subsequently determined. **(F, G)** Immunoblot analysis and quantification of BAX and Bcl2 in HS-treated LO2 cells. **(H)** Apoptosis was measured using Annexin V-FITC/PI staining. Data are shown as the mean ± SD. n = 4. *p < 0.05.

Next, to further assess the regulation of Parkin-mediated mitophagy by p53 in LO2 cells following HS, a specific adenovirus was used Rya Marasiganto achieve p53 overexpression (**Figure S4E**). p53 overexpression strongly inhibited HS‐induced translocation of Parkin from the cytoplasm to the mitochondria (**Figure 6A**). Furthermore, p53 overexpression markedly suppressed expression levels of mitophagy-related proteins in LO2 cells following HS (**Figure 6B**). p53 overexpression also decreased VDAC ubiquitination levels and formations of mitophagosomes (**Figures 6C, D**). Moreover, p53 overexpression significantly increased hepatocyte apoptosis (**Figures 6E–H**).

**Figure 7:**
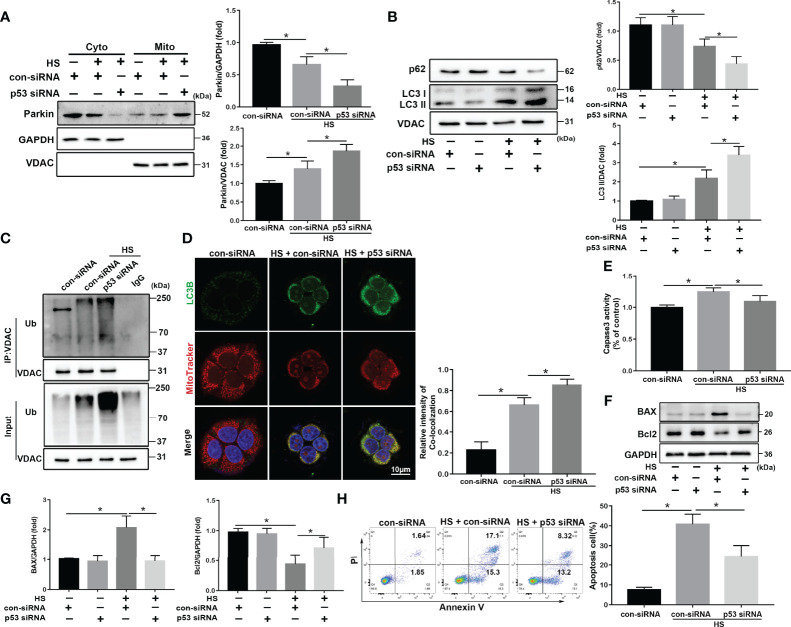
p53 knockdown attenuates apoptosis through Parkin-dependent mitophagy *in vitro*. After transfection with con-siRNA or p53 siRNA for 8 h, LO2 cells were exposed to 42°C for 3 h for HS treatment followed by incubation at 37°C for 6 h **(A)** Immunoblot analysis and quantification of Parkin in the cytoplasm and mitochondrial fractions after HS. **(B)** Immunoblot analysis and quantification of autophagy-related proteins p62 and LC3-II in the mitochondrial fractions of LO2 cells. **(C)** Immunoprecipitation analysis of VDAC ubiquitination levels. **(D)** Representative images and quantification of immunofluorescence double-labeling LC3B (green) and MitoTracker stains (red) in HS-treated LO2 cells. Scale bar: 10 μm. **(E)** The enzymatic activity of Caspase 3 was subsequently assessed. **(F, G)** Immunoblot analysis and quantification of BAX and Bcl2 in HS-treated LO2 cells. **(H)** Apoptosis was measured using Annexin V-FITC/PI staining. Data are shown as the mean ± SD. n = 4. *p < 0.05.

We speculated that p53 deletion would enhance Parkin-mediated mitophagy activation in LO2 cells following HS. To test this hypothesis, p53 siRNA was used to inhibit p53 expression in LO2 cells (**Figure S4F**). As expected, knockdown of p53 markedly promoted HS‐induced translocation of Parkin from the cytoplasm to the mitochondria (**Figure 7A**) and significantly enhanced mitophagy activation in HS-treated LO2 cells (**Figures 7B–D**). In addition, p53 deletion also inhibited HS-induced apoptosis in HS-treated LO2 cells (**Figures 7E–H**). Taken together, these results suggest that Parkin-dependent mitophagy and apoptosis are activated by HS, which are further exacerbated by p53 overexpression in HS-treated LO2 cells, whereas inhibition of p53 may effectively reverse this process.

### p53 Regulates Mitophagy and Mitochondrial Damage *via* Parkin

**Figure 8:**
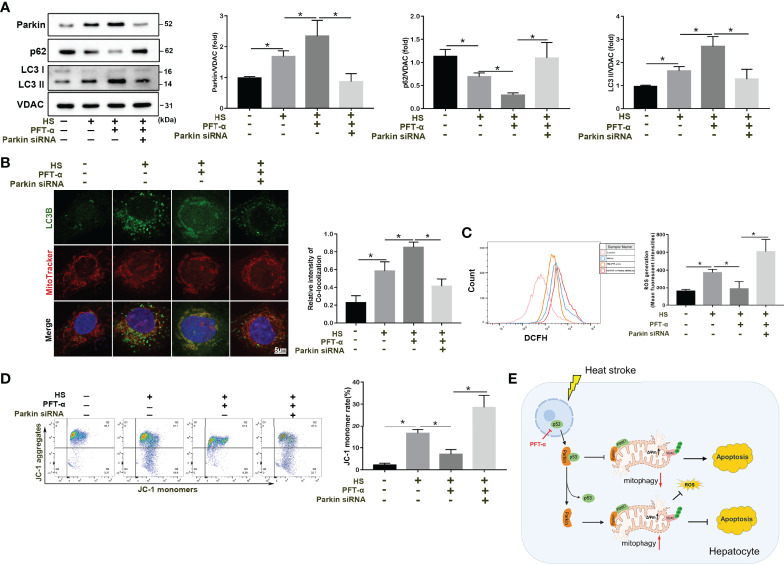
p53 regulates mitophagy and mitochondrial damage through Parkin. After transfection with con-siRNA or p53 siRNA for 8 h, LO2 cells were pretreated with the p53 inhibitor PFT-α (20 µM) and then subjected to HS. **(A)** Immunoblot analysis and quantification of mito/autophagy-related proteins, including Parkin, p62, and LC3-II, in the mitochondrial fractions of LO2 cells. **(B)** Representative images and quantification of immunofluorescence double-labeling LC3B (green) and MitoTracker stains (red) in HS-treated LO2 cells. Scale bar: 5 μm. **(C)** Representative images and quantification of the ROS probe DHE staining in HS-treated LO2 cells. **(D)** Flow cytometry analysis of ΔΨm in LO2 cells stained with JC-1. **(E)** Proposed mechanism by which p53-Parkin-mediated mitophagy alleviates hepatocyte apoptosis in HS-ALI. In response to HS, increased translocation of p53 from the nucleus to the cytoplasm is induced. Subsequently, cytosolic p53 binds to Parkin, which blocks Parkin mitochondrial translocation and reduces VDAC ubiquitination levels, leading to inhibition of mitophagy, upregulation of ROS production, and ultimately exacerbating hepatocyte apoptosis. Of note, p53 deficiency effectively reverses this process. Data are shown as the mean ± SD. n =4. *p < 0.05.

We subsequently explored the molecular mechanisms by which p53 regulates mitophagy and oxidative stress *via* Parkin. p53 inhibitor PFT-α significantly enhanced mitophagy activation in LO2 cells following HS (**Figure 8A**). Consistent with the immunoblotting results, colocalization of LC3B and MitoTracker demonstrated increased formations of mitophagosomes (**Figure 8B**). However, the protective effect of PFT-α was eliminated in Parkin siRNA-treated LO2 cells (**Figures 8A, B**). Similar results were also observed with respect to mitochondrial damage as assessed by flow cytometry analysis. As shown in **Figures 8C, D**, PFT-α also upregulated mitochondrial ΔΨm and decreased ROS production, which were effectively reversed by silencing Parkin. Overall, these results demonstrate that PFT-α treatment ameliorates mitochondrial damage through Parkin-mediated mitophagy in HS-induced LO2 cells.

## Discussion

It is well known that mitophagy, the principal mechanism to eliminate impaired mitochondria, is important for the regulation of mitochondrial function and cellular homeostasis (32, 33). Here, our results indicated an increase of PINK1 expression levels in the mitochondria at 6 h after HS, and an increase in the translocation of Parkin from the cytoplasm to the mitochondria *in vivo* and *in vitro*, suggesting that HS treatment stabilized PINK1 at the outer mitochondrial membrane and thereby recruits more Parkin to damage mitochondria. Interestingly, Recent evidence has suggested a linear PINK1-Parkin mitophagy pathway, which places PINK1 as a critical upstream molecule for Parkin recruitment (34). PINK1-mediated phosphorylation recruits the E3 ubiquitin ligase Parkin, leading to ubiquitination of substrates on damaged mitochondria (35). Meanwhile, one recent study demonstrated the protective role of Parkin-mediated mitophagy in Contrast-induced acute kidney injury (CI-AKI) (36). Parkin-mediated mitophagy was also indicated that contribution to liver pathophysiology (37). Our data also demonstrated that Parkin-mediated mitophagy deficiency may exacerbate HS-induced hepatocyte apoptosis by using Parkin siRNA. Thus, Parkin also plays an important role in the induction of mitophagy. In addition, we observed that mitochondrial dysfunction was necessary for cellular injury or death in response to HS. Meanwhile, although both hepatocyte apoptosis and mitophagy increased at 0-6 h after HS treatment, mitophagy deficiency exacerbated hepatocyte injury and apoptosis after HS, supporting a protective role of mitophagy in HS-ALI. Moreover, we demonstrated cytosolic p53 inhibited Parkin’s mitochondrial translocation, preventing the removal of damaged mitochondria and exacerbating hepatocyte apoptosis and oxidative stress. These findings provide a mechanistic explanation for the observed HS-induced compromise of mitochondrial integrity and functional decline in HS-ALI.

Emerging evidence indicates that mitophagy plays a crucial role in the regulation of liver homeostasis (38, 39). Impaired mitophagy is known to contribute to several liver pathologies, including drug-induced liver injury, hepatic ischemia-reperfusion injury (IR), alcoholic liver disease (ALD), nonalcoholic fatty liver disease (NAFLD), and viral hepatitis (40). In IR-ALI, Xu et al. found that increased hepatic mitophagy is induced at the early stages of IR, both *in vivo* and *in vitro*, which is linked to elevated cell death and aggregated liver injury (41). In drug-induced liver injury, acetaminophen (APAP) administration enhances Parkin’s mitochondrial translocation and concurrently mitophagy induction in mouse livers (42). However, the role of mitophagy in HS-AL remains unknown. Notably, we provide additional mechanistic evidence that Parkin-mediated mitophagy was activated in hepatocytes exposed to HS-ALI, as illustrated by Parkin deletion, Mdivi-1, and 3-MA treatment. Our data demonstrated that defective Parkin-mediated mitophagy resulted in excessive accumulation of damaged mitochondria, leading to ROS generation and ΔΨm depolarization. Inhibition of mitophagy resulted in increased levels of apoptosis and mitochondrial injury in hepatocytes following HS, indicating a protective role of mitophagy in HS-ALI by restraining apoptosis and mitochondrial injury in hepatocytes.

With respect to the regulatory pathways that influence mitochondrial integrity and function, we found that p53 was potently activated in HS-treated hepatocytes. Growing evidence suggests that p53 is a transcription factor that is activated by multiple different stresses, including DNA damage, oncogene activation, oxidative stress, and disruption of nucleolar function (43, 44). In addition, p53 exerts differential effects on cellular function according to its cellular concentration and distribution. Genotoxic stress provokes p53 accumulation in both the nucleus and cytosol, and endogenous cytosolic p53 directly activates BAX to induce apoptosis (45). p53 is also known to restrict autophagy activation through a cytosolic effect (46). The direct molecular link between p53 activation and Parkin-mediated mitophagy deficiency contributes to the mitochondrial compromise associated with aging-related and doxorubicin (DOX)-mediated decreases in cardiac contractility (47). Here, we found that p53 translocation from the nucleus to the cytoplasm was increased by HS in a time-dependent manner. The endogenous Parkin-p53 complex was identified in immunoprecipitants of endogenous Parkin and p53 in the cytosolic lysate, as well as colocalization of p53 and Parkin indicated by immunofluorescence staining, indicating that cytosolic p53 binds to Parkin in HS-treated LO2 cells. Consistent with previous studies, our results also demonstrated that p53 inhibits mitophagy by inhibiting Parkin’s translocation from the cytosol to mitochondria, which in turn reduces VDAC ubiquitination levels. Previous studies demonstrated that p53 can bind to the RING0 region of Parkin, disrupting Parkin’s biological function and affecting damaged mitochondrial clearance and cellular redox homeostasis (28, 48). Whether this also occurs in HS-treated hepatocytes requires further study.

Although these findings demonstrated the key role of p53 in Parkin-dependent mitophagy, to the best of our knowledge, the exact mechanism whereby p53 controls mitochondrial homeostasis remains unclear. In particular, previous studies have demonstrated that p53 regulates mitochondrial homeostasis through nuclear, cytosolic or intramitochondrial sites of action (49, 50). A recent study demonstrated that nuclear p53 decreases the specialized autophagy-related mitophagy response by transcriptionally downregulating PINK1 (51). Consistently, the p53-dependent anti-autophagic phenotype exclusively accounts for cytosolic p53 in sepsis-induced acute kidney injury, which may promote proteasomal degradation of the autophagic protein Beclin1 (52). p53 preserves mitochondrial biogenesis and function in the setting of the telomeric DNA damage response (DDR) (53). Of note, the mechanism underlying the role of p53 on different cellular distributions in regulating mitochondrial homeostasis remains unclear, and further studies are needed to address these gaps in knowledge. In this study, p53 overexpression using a specific adenovirus or Tenovin-6 exacerbated hepatocyte apoptosis and oxidative stress through habiting Parkin-dependent mitophagy in HS-ALI, whereas inhibition of p53 using siRNA or PFT-α effectively reversed this process. Consequently, it appears certain that the mitochondria-related functions of p53 have broader implications than previously thought in HS-ALI, providing new therapeutic targets for treatment.

In summary, our present study provides a molecular understanding of Parkin-dependent mitophagy in HS-ALI. We uncovered a novel concept, as shown in **Figure 8E**, whereby increased cytosolic p53 inhibits Parkin-mediated mitophagy and provokes mitochondrial compromise in response to HS-ALI. In particular, the enhancement of p53 activation and Parkin-mediated mitophagy deficiency aggravated apoptosis and ROS production following HS. And p53 deficiency effectively with inhibitor reverses this process. Therefore, our study shows that p53 has a previously undescribed pathogenic effect on HS-treated hepatocytes in which cytosolic p53 aggravates apoptosis by inhibiting Parkin-mediated mitophagy. p53‐parkin‐mediated mitophagy may represent a novel target for attenuating HS-ALI.

## Data Availability Statement

The raw data supporting the conclusions of this article will be made available by the authors, without undue reservation.

## Ethics Statement

The animal study was reviewed and approved by Ethical Committee for Animal Experimentation of Nanfang Hospital.

## Author Contributions

WH and WX were involved in conception and design, performance of experiments, and composition of this manuscript. HZ and SC performed all the experiments and analyzed our results. YoL, QH, and YaL were involved in the data acquisition. YaL and ZZ confirmed the authenticity of the raw data. All authors analyzed the data, and reviewed and approved the final manuscript.

## Funding

This study was supported by grants from the National Natural Science Foundation of China (81701955, 81871604, and 82172181).

## Conflict of Interest

The authors declare that the research was conducted in the absence of any commercial or financial relationships that could be construed as a potential conflict of interest.

## Publisher’s Note

All claims expressed in this article are solely those of the authors and do not necessarily represent those of their affiliated organizations, or those of the publisher, the editors and the reviewers. Any product that may be evaluated in this article, or claim that may be made by its manufacturer, is not guaranteed or endorsed by the publisher.
