# Supplementary material for: Cytosolic p53 Inhibits Parkin-Mediated Mitophagy and Promotes Acute Liver Injury Induced by Heat Stroke
Source: Front Immunol. 2022 May 13;13:859231. doi: 10.3389/fimmu.2022.859231 (PMC9139682; doi:10.3389/fimmu.2022.859231)
Supplement: Supplementary file 1 [file DataSheet_1.doc]

**Supplemental Information**

**Supplementary Figure 1.**


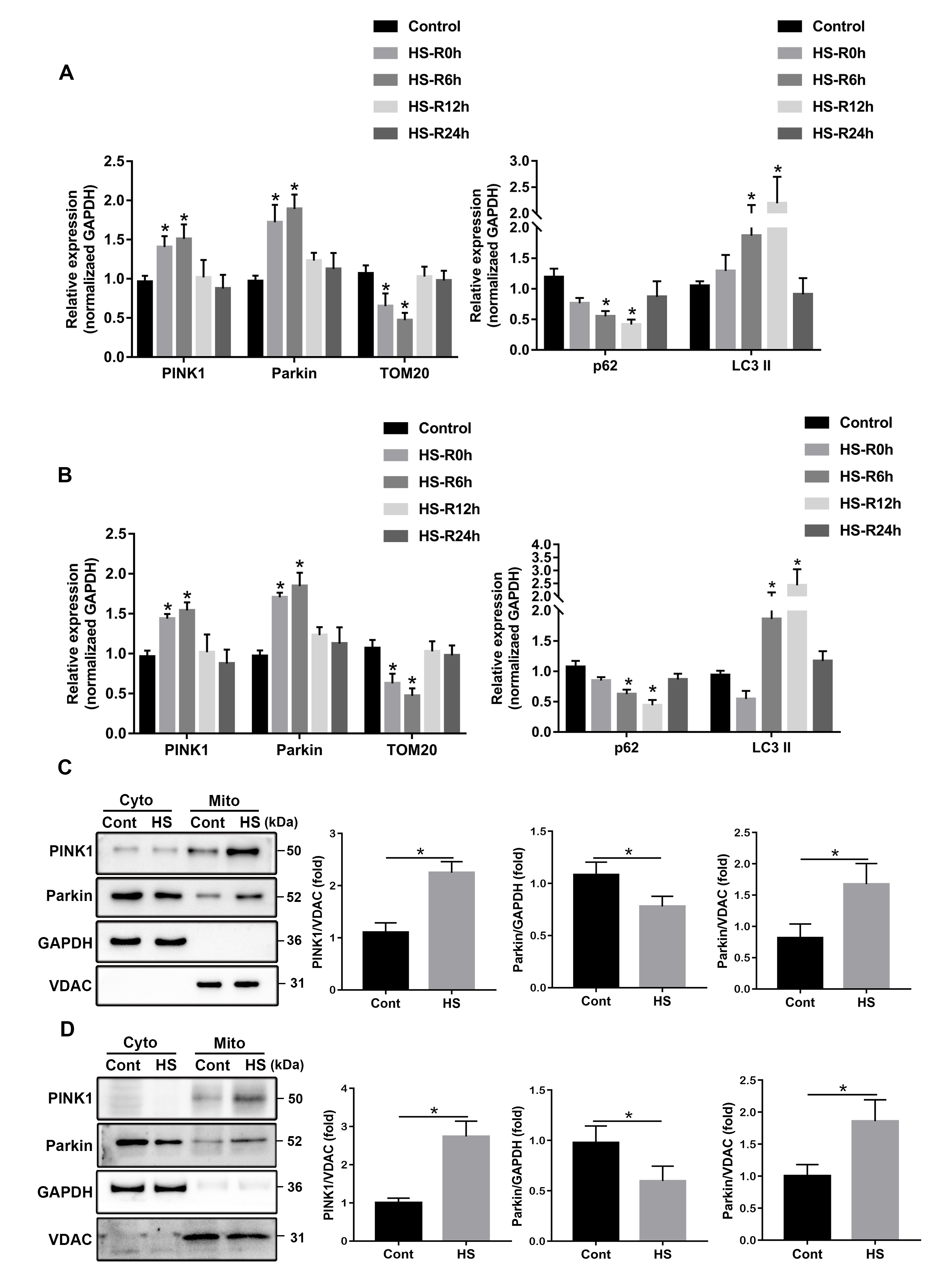


**FIGURE S1 |** Mitophagy is induced by HS in vivo and in vitro. **(A)** Immunoblot analysis and quantification of mitophagy-related proteins, including PINK1, Parkin, TOM20, LC3 II, and p62 in liver tissues. **(B)** Immunoblot analysis and quantification of mitophagy-related proteins, including PINK1, Parkin, TOM20, p62, and LC3 II in LO2 cells. **(C)** Liver tissues were obtained from HS and sham control mice at 6 h after HS. Immunoblot analysis and quantification of the mitophagy-related proteins PINK1 and Parkin in the cytoplasm and mitochondrial fractions of livers, n=3-4. **(D)** LO2 cells were exposed to 42°C for 3 h for HS treatment and then incubated at 37°C for 6 h later. Immunoblot analysis and quantification of the mitophagy-related proteins PINK1 and Parkin in the cytoplasm and mitochondrial fractions of LO2 cells, n=3-4. Data are shown as the mean ± SD. *p < 0.05.

**Supplementary Figure 2.**


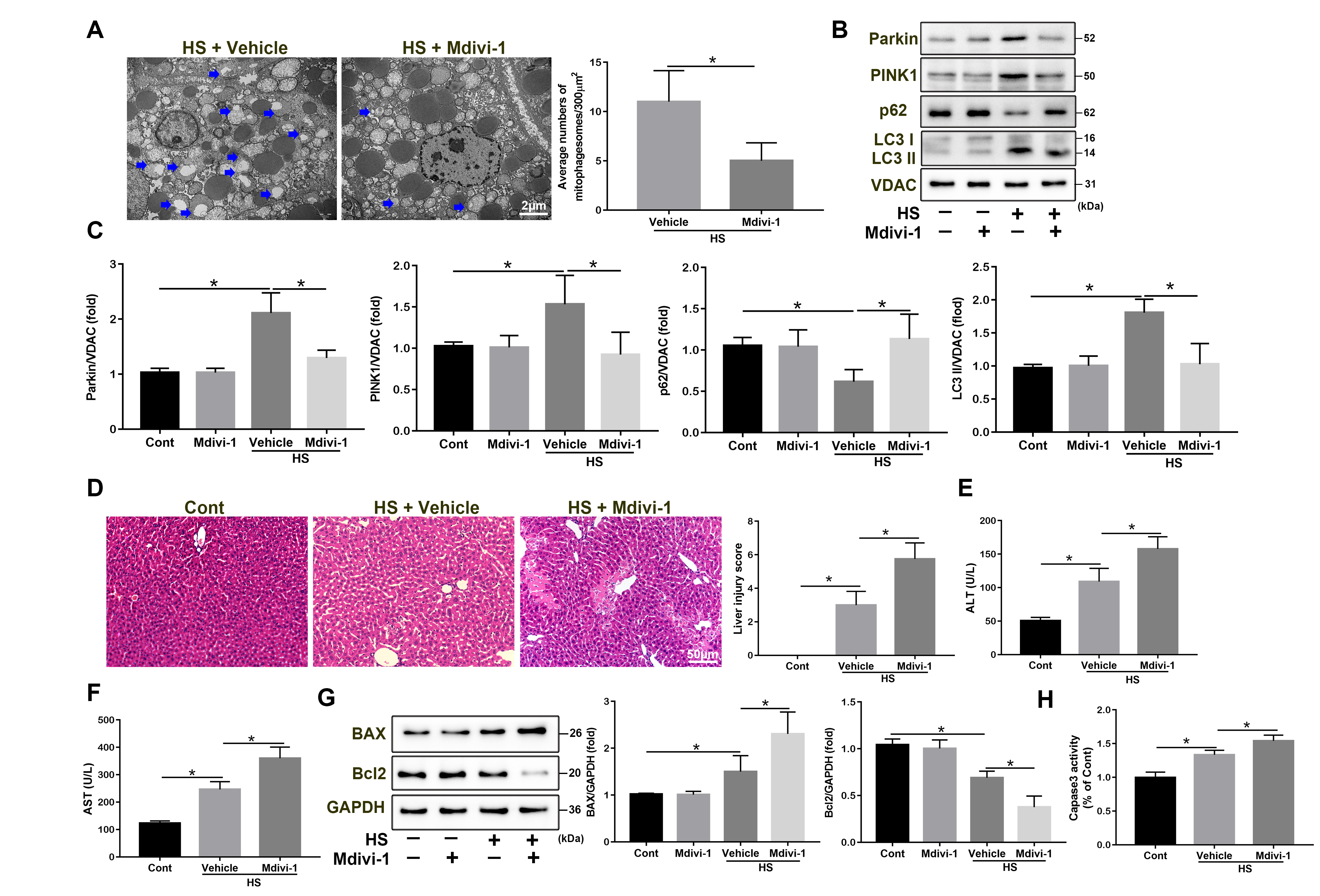


**FIGURE S2 |** Midiv-1 treatment increases apoptosis in HS-ALI by inhibiting mitophagy. Midiv-1 treatment increases apoptosis in HS-ALI by inhibiting mitophagy. Mice were pretreated with Mdivi-1 (25 mg/kg, i.p.) for 2 h and then subjected to sham-untreated or HS. Livers and [serum](http://dict.cn/blood serum) were collected 6 hours after HS. **(A)** Representative TEM images of mitochondrial morphology in hepatocytes after HS. Blue arrows: mitophagosome and mitolysosome. Scale bar: 2 μm. **(B-C)** Immunoblot analysis and quantification of mito/autophagy-related proteins, including Parkin, PINK1, p62, and LC3 II in the mitochondrial fractions of livers. **(D)** Representative histology and pathological score of liver samples by H&E staining. Scale bar: 50 μm. **(E-F)** Relative serum ALT and AST levels. **(G)** Immunoblot analysis and quantification of BAX and Bcl2 in liver tissues. **(H)** The enzymatic activity of Caspase 3 was subsequently measured, n=4. Data are shown as the mean ± SD. *p < 0.05.

**Supplementary Figure 3.**


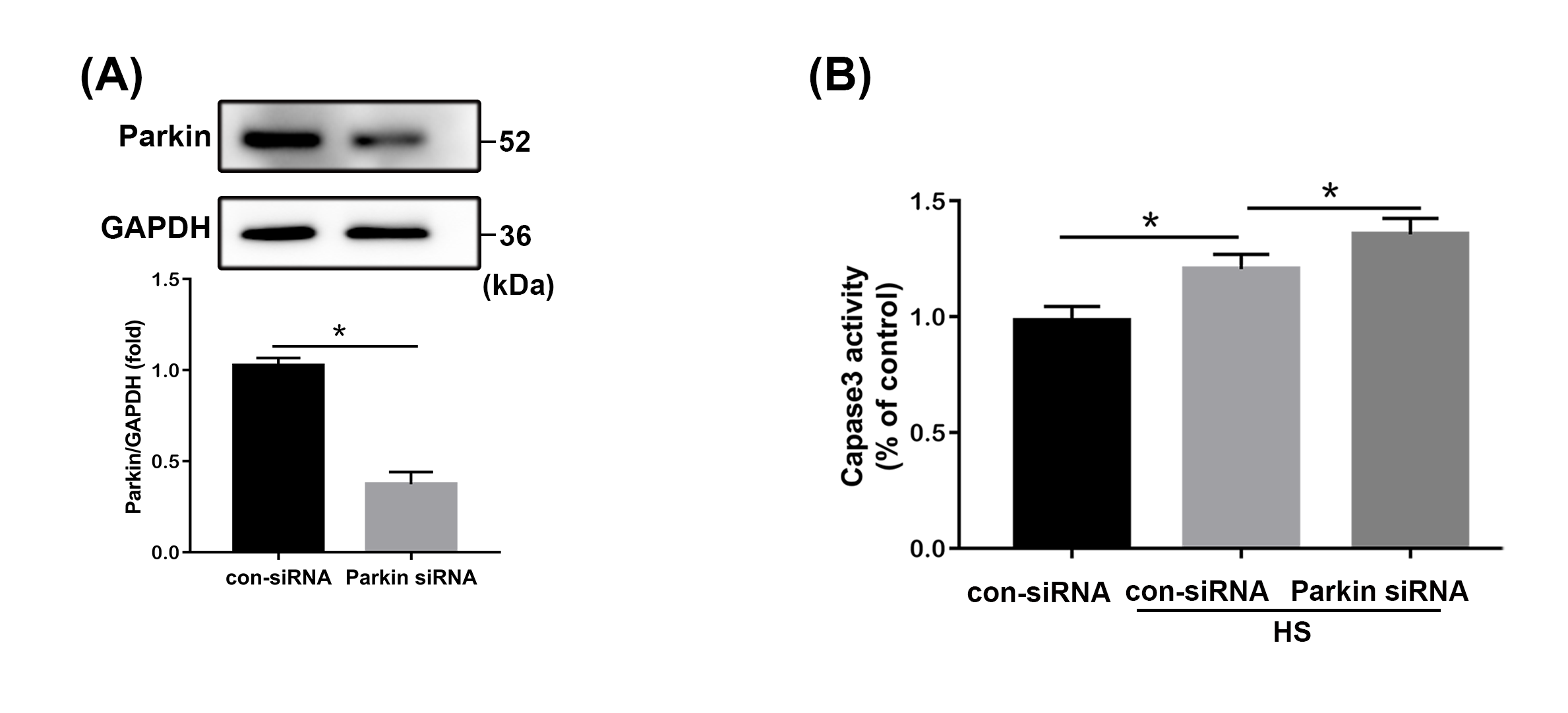


**FIGURE S3 |** Confirmation of Parkin siRNA-mediated Parkin knockdown. After transfection with con-siRNA or Parkin siRNA for 8 h, LO2 cells were exposed to 42 °C for 3 h and then incubated at 37°C for 6 h. **(A)** Confirmation of Parkin siRNA-mediated Parkin knockdown by western blot. **(B)** The enzymatic activity of Caspase 3 was measured, n=4. Data are shown as the mean ± SD. *p < 0.05.

**Supplementary Figure 4.**


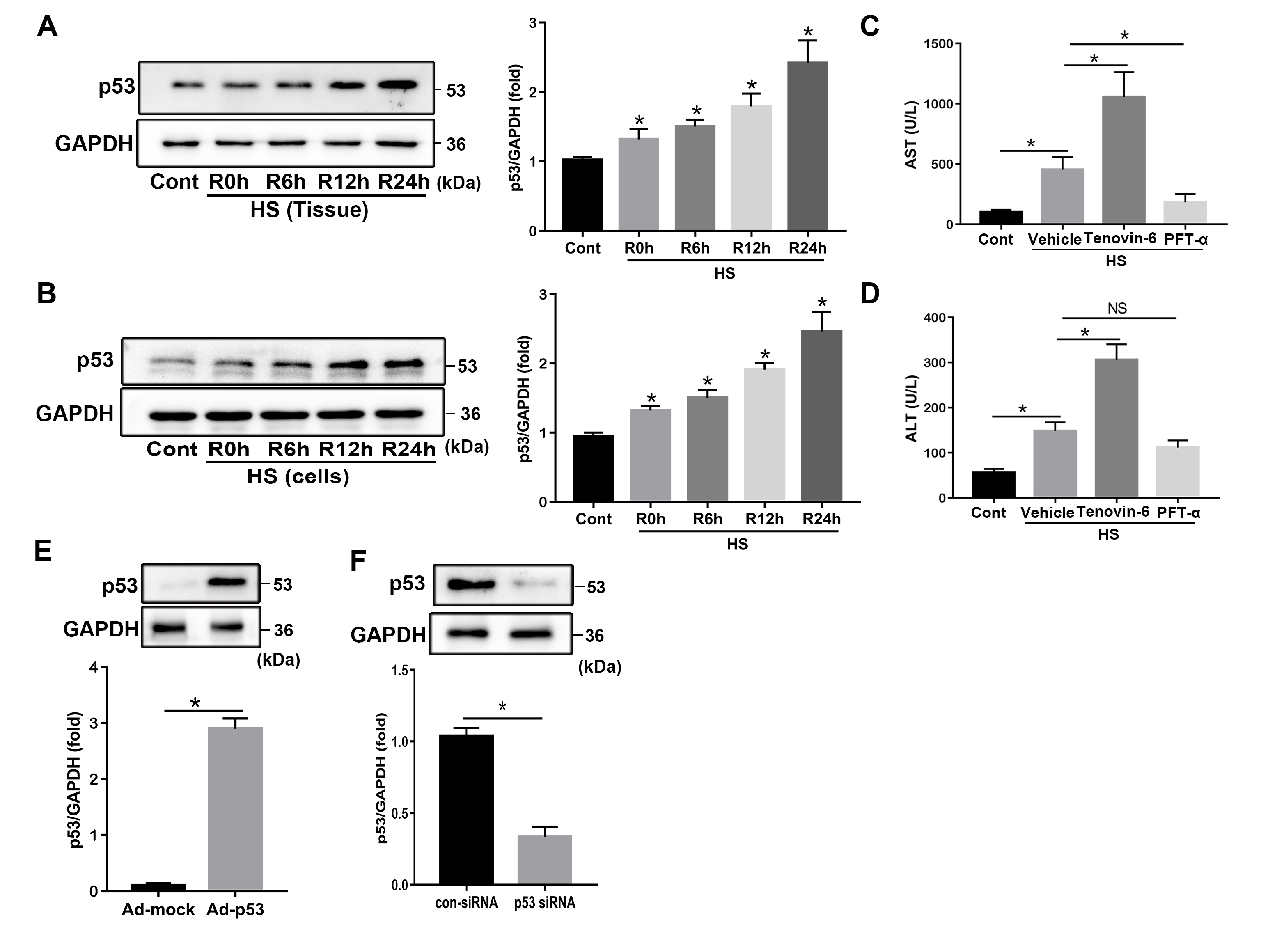


**FIGURE S4 |** p53 gradually increased following HS in vivo and in vitro. Confirmation of adenovirus-induced p53 overexpression and siRNA-induced p53 silencing. **(A)** LO2 cells were exposed to 42°C for 3 h for HS treatment and then incubated at 37°C for 0 h and 6 h, 12 h, and 24 h later. Immunoblot analysis and quantification of p53 in liver tissues. **(B)** Immunoblot analysis and quantification of p53 in HS-treated LO2 cells. **(C, D)** p53 regulates Parkin-dependent mitophagy and apoptosis in HS-ALI. Mice were pretreated with the selective p53 agonist Tenovin-6 (25 mg/kg, i.p.) or the p53 inhibitor PFT-α (2.2 mg/kg, i.p.) for 2 h and then subjected to HS, these livers and [blood](http://dict.cn/blood serum) were collected 6 hours after HS. Relative serum ALT and AST levels. **(E)** Confirmation of the effect of adenovirus-induced p53 overexpression using western blot. **(F)** Confirmation of the efficiency of p53 silencing by western blot, n=4. Data are shown as the mean ± SD. *p < 0.05, NS = not significant.
